# Polycyclic Aromatic Hydrocarbons and Mammary Cancer Risk: Does Obesity Matter too?

**Published:** 2021

**Authors:** Lydia Lichtiger, Janelle Rivera, Debashish Sahay, Rachel L. Miller

**Affiliations:** Division of Clinical Immunology, Department of Medicine, Icahn School of Medicine, Mount Sinai, New York City, NY, United States

**Keywords:** Polycyclic aromatic hydrocarbons, Breast cancer risk, Estrogen receptor α, Obesity, Environmental exposures, Breast cancer gene 1, Aryl hydrocarbon receptor

## Abstract

Breast cancer risk remains incompletely explained, and higher incidence rates of breast cancer over recent times and in urban and industrialized areas suggest environmental causes. Polycyclic aromatic hydrocarbons (PAH) are ubiquitous in the environment and epidemiological and rodent studies have shown associations between exposure to PAH and breast cancer incidence as well as mammary tumorigenesis. In addition, *in vitro* and rodent studies have implicated alterations in estrogen receptor alpha (*Erα*) signaling pathways following PAH exposure in limited experimental studies. However, our understanding of these mechanisms is incomplete. Sahay et al. addressed this gap by examining the effect of PAH exposure on epigenetic and transcriptional regulation of genes in the *Erα* pathway in a mouse cohort exposed to aerosolized PAH at proportions measured in urban air. In addition to alterations in the *Erα* signaling pathway in the pregnant mice and in their offspring and grandoffspring, the investigators observed higher body weights in mice exposed to PAH compared to the control. Given that associations between mammary tissue adiposity, systemic adiposity, and breast cancer risk have been observed previously, the finding of higher body weight in the PAH exposure group raises the possibility that body weight might influence the association between PAH exposure and breast cancer risk. Along with new analyses, we discuss the possibility that body weight may modify the association between PAH exposure, mammary cellular proliferation, and mammary gland ductal hyperplasia in offspring and grandoffspring mice and future research that may be needed to delineate these associations.

Breast cancer is the most commonly diagnosed cancer in women in the United States [1]. Breast cancer incidence rates, particularly among younger women, have been increasing since the 1930s, and are only partially explained by changes in parity, age at first birth, and improved screening and detection [2,3]. Many questions remain unanswered regarding the mechanisms that underlie current risk.

Emerging evidence indicates that environmental exposures contribute to current breast cancer risk. The proportion of breast cancer risk attributable to traditional risk factors, such as weight, height, age at menarche, parity, age at first birth, duration of breastfeeding, menopausal status, menopausal hormone therapy, family history of breast cancer, history of benign breast biopsy, greater breast density (the ratio of dense fibroglandular tissue to nondense adipose tissue), alcohol consumption, and physical activity, account for 53 – 70% of breast cancer incidence [4,5]. Risk also varies across geographical regions and by urbanicity [6–8]. These findings, plus observations about rising incidence rates of breast cancer that cannot be attributed to changes in the gene pool [2,3], implicate a substantial role for the environment.

Studies of the importance of ambient air pollution to breast cancer risk have been inconsistent [9,10]. The emerging evidence, however, implicates a class of air pollutants that disrupt the endocrine system in breast cancer risk [10]. These are the polycyclic aromatic hydrocarbons (PAH), that are formed during incomplete combustion of organic fuels and are ubiquitous in the environment [11]. Previously, individual PAH compounds have been classified as carcinogenic, probably carcinogenic, and possibly carcinogenic to humans [12].

Many studies, though not all, have shown epidemiological associations between measures of PAH or surrogates of PAH exposure, and the development of breast cancer. For example, in the Long Island Breast Cancer Study Project (LIBCSP), a population-based case-control study of mostly postmenopausal women, the odds of breast cancer were elevated 44% in association with long-term vehicular traffic benzo[13]pyrene (B[12]P) levels in the top 5% (vs below the median) estimated during the period of 1960–1990, and were elevated 14% for one year recent exposure in 1995 [13]. In the same cohort, ever burning synthetic logs indoors, another surrogate for inhaled PAH exposure, was associated with a 42% increased risk of breast cancer [14]. Breast cancer incidence also has been shown to be elevated 32% in women who are suspected to have occupational exposure to PAHs, estimated from lifetime work history and based on Occupational Safety and Health Administration chemical exposure data in industries that include chemical manufacturing, electrical equipment, appliance, and component manufacturing, and building construction compared to women without occupational exposure to PAHs [15]. Other epidemiological studies assessed the association between grilled, smoked, and charred meat intake, dietary sources of PAHs, and breast cancer with contrasting results. In two case control studies, the odds of breast cancer were elevated 16% [16] and 20% [17] in association with self-reported grilled/smoked/roasted meat consumption, a surrogate of PAH exposure. An additional case control study also found higher odds of breast cancer in association with self-reported consumption of red meat cooked at high temperatures, but no association was observed for estimated meat derived B[a]P [18], possibly calling into question the accuracy of using this surrogate for dietary PAH. In sum, most epidemiological studies have relied on measuring sources of PAH exposure indirectly and/or using self-reports, contributing to inconclusive understanding of the exposure and outcome relationships and possibly clouding the interpretation of most studies to date.

A few mechanistic studies in cell lines have addressed the associations between PAH exposure and breast cancer risk. In one, in MCF-7 cells, an estrogen receptor (*ER*) positive breast cancer cell line, that were treated with 5 μM of B[a]P for 24 hours, expression of the tumor suppressor breast cancer gene (*Brca*) 1 was downregulated and protein expression of the tumor suppressor gene *P53* was upregulated [19]. In MCF-7 cells transfected with a plasmid that contained a cassette that encoded a mutation in the *P53* gene and treated with 5 μM of B[a]P for 24 hours, B[a]P administration did not lower *Brca1* transcription [19]. This finding suggests B[a]P mediated downregulation of *Brca1* occurs through *P53* [19]. Thus, one way that PAHs may be involved in breast carcinogenesis is through inhibition of *Brca1* mutation repair mechanisms [19]. In another *in vitro* study, treatment for 24 hours with 6 nM of a mixture of 36 PAH compounds derived from sediment from an EPA superfund site raised aryl hydrocarbon receptor (*Ahr*) and *Erα* protein levels and increased cell proliferation in MCF-7 cell lines [20]. These findings suggest that PAH mediated upregulation of *Ahr* may cause downstream upregulation of *Erα*, resulting in cellular proliferation, a phenotype previously associated with breast carcinogenesis [21].

Rodent studies have demonstrated that PAHs delivered to animals can induce mammary tumors [12]. For example, in Sprague-Dawley rats treated by oral gavage with 10 mg of 7,12-Dimethylbenz[a]anthracene (DMBA) at age 50 days, the age of puberty onset [22], *Ahr* mRNA expression was raised in peritumoral and tumor mammary tissue compared to mammary tissue of rats treated with the negative control [23]. In addition, *Brca1* CpG promoter methylation was raised, and *Brca1* and *Erα* mRNA expression were lowered in peritumoral and tumor mammary tissue compared to control rats [23]. Together, these findings support previous observations that activation of the *Ahr* by PAH compounds are involved in the suppression of *Brca1*, possibly through altered DNA methylation [24]. In addition, because *Erα* and *Brca1* have been shown to upregulate the expression of the other in a reciprocal manner [25], the finding that DMBA downregulates *Brca1* and *Erα* expression suggests that DMBA may inhibit the positive feedback loop between *Brca1* and *Erα*, resulting in their coinciding suppression. Additional coupled *in vivo* and *in vitro* studies have looked at the role of *NR2E3*, a nuclear receptor that maintains normal *Erα* expression by binding to and modifying histone acetylation in its promoter region, in PAH mediated changes to *Erα* expression [26]. In MCF-7 cells treated with 5 μM of B[a]P for 90 minutes, *NR2E3* activity was decreased in the *Erα* promoter, accompanied by lower histone acetylation markers [27]. To assess whether these *in vitro* findings were maintained *in vivo*, the investigators conducted an analogous experiment in which mice were injected intraperitoneally with 125 mg / kg of B[a]P 96 hours and 48 hours prior to sacrifice [27]. *NR2E3* and *Erα* mRNA and protein levels were lowered in the livers of mice in the B[a]P exposure group compared to the control [27]. Further, the levels of *NR2E3* and *H3K4me2*, a histone acetylation marker, were lowered in the *Erα* promoter region in the livers of mice exposed to B[a]P compared to the control [27]. This demonstrated that exposure to B[a]P suppressed *Erα* expression through epigenetic modification by *NR2E3 in vitro* and *in vivo* [27].

The cell line and rodent studies described here suggest that PAH exposure modifies epigenetic and transcriptional regulation of genes in the *Erα* pathway, with implications for mammary tumorigenesis. Specifically, PAH was found to upregulate mRNA expression of the transcription factor *Ahr* [20,23], presumably through ligand binding [28], with conflicting downstream effects on *Erα* mRNA expression, including both raised [20] and lowered [23,27] expression. Given that PAH compounds have been shown to exert anti-estrogenic [29,30] and pro-estrogenic effects [31,32], this discrepancy likely can be attributed to differences in the estrogenic activities of the PAH compounds used in these studies. Metabolites of B[a]P, which were used in Sahay et al. [33], naphthalene, phenanthrene, used in Gearhart-Serna et al. [20], and pyrene, which was used in both studies, additionally have been shown to have anti-estrogenic and pro-estrogenic effects through *Erβ* [30,34]. Despite the conflicting findings of the effect of PAH compounds on *Erα* expression in the three studies described here, PAH mediated anti-estrogenic and pro-estrogenic effects are associated with cell proliferation [30,35,36], which suggests that both effects may be important in malignant transformation of mammary cells through mechanisms that need further elucidation.

As only a few studies have coupled observations of the association between PAH exposure and mammary tumorigenesis with underlying mechanisms *in vivo*, the study by Sahay et al. [33] stands out. This research gap is addressed in part by pairing previous observations about the importance of *Erα* signaling in mammary carcinogenesis [20,23,27] with other previous observations about the importance of environmental epigenetic regulation [37]. PAH exposure during pregnancy was hypothesized to alter the DNA methylation and mRNA expression of genes in the *Erα* pathway in pregnant mice (F0) who were exposed to ambient PAH, as well as in the offspring (F1) and grandoffspring (F2) mice [33]. The approach was to expose pregnant mice to a mixture of 9 PAH compounds at the proportions measured in a cohort of pregnant women in New York City [38]. Sahay et al. found that PAH raised aryl hydrocarbon nuclear receptor (*Arnt*) mRNA expression and lowered *Brca1* mRNA expression in the mammary glands of the pregnant mothers at postpartum day (PPD) 28, the offspring and grandoffspring at postnatal day (PND) 60, and the grandoffspring mice at PND28 [33]. PAH also lowered aryl hydrocarbon receptor repressor (*Ahrr)* mRNA expression and raised *Ahrr* methylation at CpG sites in the mammary glands of the pregnant mothers at PPD28, the offspring and grandoffspring mice at PND60, and the offspring and grandoffspring mice at PND28 [33]. In addition, PAH raised *Erα* promoter methylation at CpG sites in the mammary glands of the pregnant mothers at PPD28 and the offspring and grandoffspring mice at PND60 and PND28 [33]. Concomitantly, PAH lowered *Erα* mRNA expression in the mammary glands of the pregnant mothers at PPD28, the offspring and grandoffspring mice at PND60, and the grandoffspring mice at PND28 [33]. PAH-induced changes in transcriptional regulation in mammary gland tissue occurred in the absence of changes in systemic gene expression [33]. In addition, changes in *Erα* and *Ahrr* mRNA expression and methylation for some CpG sites in the mothers predicted transcriptional and epigenetic regulation of *Era* and *Ahrr* in the offspring and grandoffspring mice at PND60 [33]. This finding suggests that alterations in epigenetic and transcriptional regulation of some genes in the *Erα* pathway observed in the mothers may drive the changes observed in offspring and grandoffspring mice [33].

The findings in Sahay et al. are consistent with PAH ligand binding to *Ahr* [39], dimerizing with *Arnt* [40], and downstream downregulation of *Brca1* and *Erα* mRNA expression. Consistent with Jeffy et al. [19] and Romagnolo et al. [23], exposure to PAH downregulated *Brca1*, presumably through regulation of *Ahr*. The finding of upregulation of *Erα* methylation and downregulation of *Erα* transcription contrasts with the findings of Gearhart-Serna et al. [20]. 8 of the 9 PAH compounds used in Sahay et al. [33] were also used in Gearhart-Serna et al. [20]. Despite significant overlap of PAH compounds in these studies, Gearhart-Serna et al. [20] used 28 additional compounds not used in Sahay et al. [33], and it is likely that the different composition of PAH compounds in Gearhart-Serna et al. [20] and Sahay et al. [33], as well as different models used (*in vivo* in mice and *in vitro* in a cell line) can explain the opposite findings of raised and lowered *Erα* expression with PAH exposure. Further, both Khanal et al. [27] and Sahay et al. [33] reported changes in the epigenetic regulation of *Erα* and resulting downregulation in *Erα* mRNA expression in response to PAH exposure, which suggests a role of epigenetic regulation in mediating PAH-induced mammary tumorigenesis [27,33]. Sahay et al. also demonstrated that the prenatal period might be an important window of susceptibility for epigenetic regulation in the pregnant mother and in the offspring and grandoffspring [33]. This is consistent with growing evidence that there are windows of susceptibility, particularly prenatal, when breast tissue grows and changes rapidly, that are most important to breast cancer risk [41].

Sahay et al. also provided evidence that PAH exposure increased the body weight of the offspring and grandoffspring mice at PND60 [33], an age that represents adulthood prior to reproductive senescence [42]. This finding is curious, as previous associations have been demonstrated between breast tissue adiposity and breast cancer risk, and between systemic adiposity and breast cancer risk. For example, greater breast adipose tissue has been inversely associated with breast cancer risk among pre and postmenopausal women [43]. Breast tissue is composed of nondense adipose tissue and dense fibroglandular tissue, and the percent breast density, a measure of the proportion of fibroglandular tissue in the breast, is a strong breast cancer risk factor [44]. In a meta-analysis of 13 cohort studies, among premenopausal women, the odds of breast cancer were elevated 37% in association with a 1 standard deviation increase in absolute dense fibroglandular tissue area and were lowered 19% in association with a 1 standard deviation increase in absolute nondense adipose tissue [43]. Among postmenopausal women, odds of breast cancer were elevated 38% in association with a 1 standard deviation increase in absolute dense fibroglandular tissue area and were lowered 21% in association with a 1 standard deviation increase in absolute nondense adipose tissue [43]. This study and others [45,46] implicate higher absolute dense fibroglandular tissue as a risk factor for breast cancer, and higher absolute nondense adipose tissue as potentially protective.

The relationship between systemic adiposity and breast cancer risk is more complex and varies by menopausal status and disease subtype. Measures of body size have been correlated positively with breast cancer risk in postmenopausal women [44,47–49] and have been correlated inversely with breast cancer risk in premenopausal women [50,51]. In addition, higher BMI during childhood has been associated with a lower risk of pre and postmenopausal breast cancer, suggesting a protective role of systemic adiposity in early life for later breast cancer risk [52].

Differences in breast cancer risk by body size are further divided by disease subtype. Breast cancers are classified by the expression of *ER*, progesterone receptor (*PR*), and human epidermal growth factor receptor (*HER*) [47]. Among premenopausal women, larger body size is inversely associated with *ER* positive breast cancer but positively associated with triple negative breast cancer, a subtype that lacks expression of *ER, PR*, and *HER* [51]. Conversely, among postmenopausal women, larger body size is associated with an increased risk of *ER* positive breast cancer and a reduced or unchanged risk of triple negative and *ER* negative breast cancer [51]. After menopause, the primary site of estrogen biosynthesis, the ovaries, slows, and adipose tissue becomes an important source of estrogen [51]. Specifically, aromatase in adipose tissue converts adrenal androgens into estrogens [51]. Thus, the increased incidence of *ER* positive breast cancer among postmenopausal people in larger bodies is thought to be caused by the production of estrogen in adipose tissue, which supports the survival of *ER* positive cancer [47]. The role of larger body size in breast cancer among postmenopausal people, though, is modest. In the Million Women cohort study, 7% of breast cancer cases in postmenopausal women were thought to be caused by overweight and obesity [49]. Even where larger body size is correlated with heightened breast cancer risk, weight is associated with a number of variables, including physical activity [53,54], weight cycling [54–59], and racial discrimination [60,61], that independently contribute to breast cancer risk, and confound the reported association between body size and breast cancer risk.

Notably, PAH exposure has been associated with higher body weight in experimental and epidemiological studies. In a mouse cohort in which pregnant mice were exposed to the same PAH mixture and levels used in Sahay et al. [33], PAH exposure was associated with higher body weight and greater fat mass in the offspring and grandoffspring mice [62]. PAH exposure during the prenatal time window also was associated with lower methylation of the peroxisome proliferator-activated receptor gamma (*Pparγ*) promoter and increased mRNA expression of *Pparγ*, *C/EBPα*, and *Cox-2*, which are transcription factors involved in adipocyte differentiation and function [62]. In a birth cohort study, prenatal PAH exposure measured through personal ambient air monitoring during the third trimester of pregnancy, that was the basis for the PAH compounds and exposure levels used in Sahay et al. [33] and Yan et al. [62], was associated with higher BMI in children at age 5 and age 7 [38]. Similarly, in a longitudinal cohort study, urinary PAH metabolite concentrations in girls at age 7 were associated with systemic adiposity, and this association continued through adolescence [63].

Studies showing an association between PAH exposure and breast cancer risk and between PAH exposure and systemic adiposity raise the question as to whether the links between air pollution or PAH specifically and breast cancer may be influenced by the presence of systemic adiposity. In new analyses, body weight in offspring and grandoffspring mice at PND60 did not differ by the extent of cellular proliferation (B coefficient = 0.19, p = 0.15, n = 58) or presence of ductal hyperplasia in mammary tissue (i.e. presence of developed tuboalveolar units lined by three or more cell layers; mean body weight without hyperplasia = 19.12 ± 0.16 g, n = 23, and with hyperplasia = 19.50 ± 0.40 g, n = 35, p = 0.77, Mann Whitney U) (Figure 1). In analyses stratified by PAH vs control exposure, in neither experimental group did body weight associate with the extent of cellular proliferation (control group B coefficient = −0.07, p = 0.72, n= 27; PAH group B coefficient = 0.18, p = 0.34, n = 31) or the presence or absence of hyperplasia (control group mean body weight without hyperplasia = 18.84 ± 0.24 g and with hyperplasia 18.06 ± 0.25 g, p = 0.10, n = 27; PAH group mean body weight without hyperplasia = 19.49 ± 0.16 g and with hyperplasia = 20.13 ± 0.50 g, p = 0.71, n = 31). Notably, the significant association between PAH exposure and cellular proliferation among offspring mice at PND60 (B coefficient = 0.41, p = 0.02, n= 31) [33] weakened to a marginal extent after new adjustment for body weight (B coefficient = 0.35, p = 0.07, n = 31). Body weight may have modified the association between PAH exposure and cellular proliferation by 15.53%. While underpowered, the results may suggest a trend for weight influencing the association between PAH exposure and cellular proliferation.

To our knowledge, only a few other studies have examined effect modification by BMI on the association between exposure to PAH and mammary or breast cancer. In the LIBCSP cohort, BMI ≥ 25kg/m^2^ strengthened the association between PAH exposure determined from self-reported grilled / smoked meat consumption and environmental tobacco smoke (ETS) exposure and breast cancer, but did not modify the association between estimated PAH exposure from active smoking or from residential vehicular traffic and breast cancer [64]. In contrast, BMI ≥ 25kg/m^2^ weakened the association between PAH exposure from self-reported indoor synthetic log burning and breast cancer [64]. Given the inconsistency of these findings, it is not clear whether BMI influences the association between PAH exposure and breast cancer risk. Additional inconsistencies become apparent when considering the possible role of PAH exposure on breast adiposity. Breast adipose tissue increases with increasing systemic adipose tissue [46] and has been shown to protect against breast cancer [43]. Thus, greater systemic adiposity may reduce premenopausal breast cancer risk through its association with breast adiposity [52]. However, PAH has opposing effects on systemic adiposity and the proportion of breast adipose tissue. Specifically, PAH has been associated with increased systemic adiposity [33,62,63] and higher breast density. In a study of five mammographic registries, the odds of dense breasts (classified as BI-RADS category 3 or 4, compared to nondense breasts classified as BI-RADS category 1 or 2) were elevated 60%, 66%, and 27% for the second, third, and fourth quartile of PAH exposure, respectively, compared to the first quartile of exposure, estimated from the 2011 EPA National Air Toxics Assessment [65]. PAH exposure may increase dense fibroglandular tissue through augmented *Erα* pathway signaling [20], which has been shown to promote proliferation of mammary fibroglandular cells [66].

Despite the apparent complicated relationship between PAH, systemic and breast adiposity, and breast cancer risk [6,24], little remains understood about the underlying mechanisms. In this context, Sahay et al. [33] helps to elucidate how altered epigenetic regulation of the *Erα* pathway and subsequent alterations in gene expression in this pathway may contribute to mammary carcinogenesis. A unique strength of this study is the focus on physiological delivery of ambient PAH over time in animals to mimic human exposure during pregnancy [33]. In addition to associations with mammary cancer risk, PAH exposure in Sahay et al. [33] also was associated with higher body weight, raising the question of whether systemic adiposity augmented the association between PAH exposure and breast cancer risk. Analysis of effect modification of the association between exposure to PAH and mammary cancer risk by body weight was underpowered and only revealed a trend. Further research into how PAH exposure, including through changes in breast density [65] and systemic adiposity [33], influences breast cancer risk will provide a more complete understanding of PAH mediated breast cancer risk. In addition, future studies may help discern key mechanisms by determining the events downstream of PAH effects on *ERα* signaling.

## Figures and Tables

**Figure 1:**
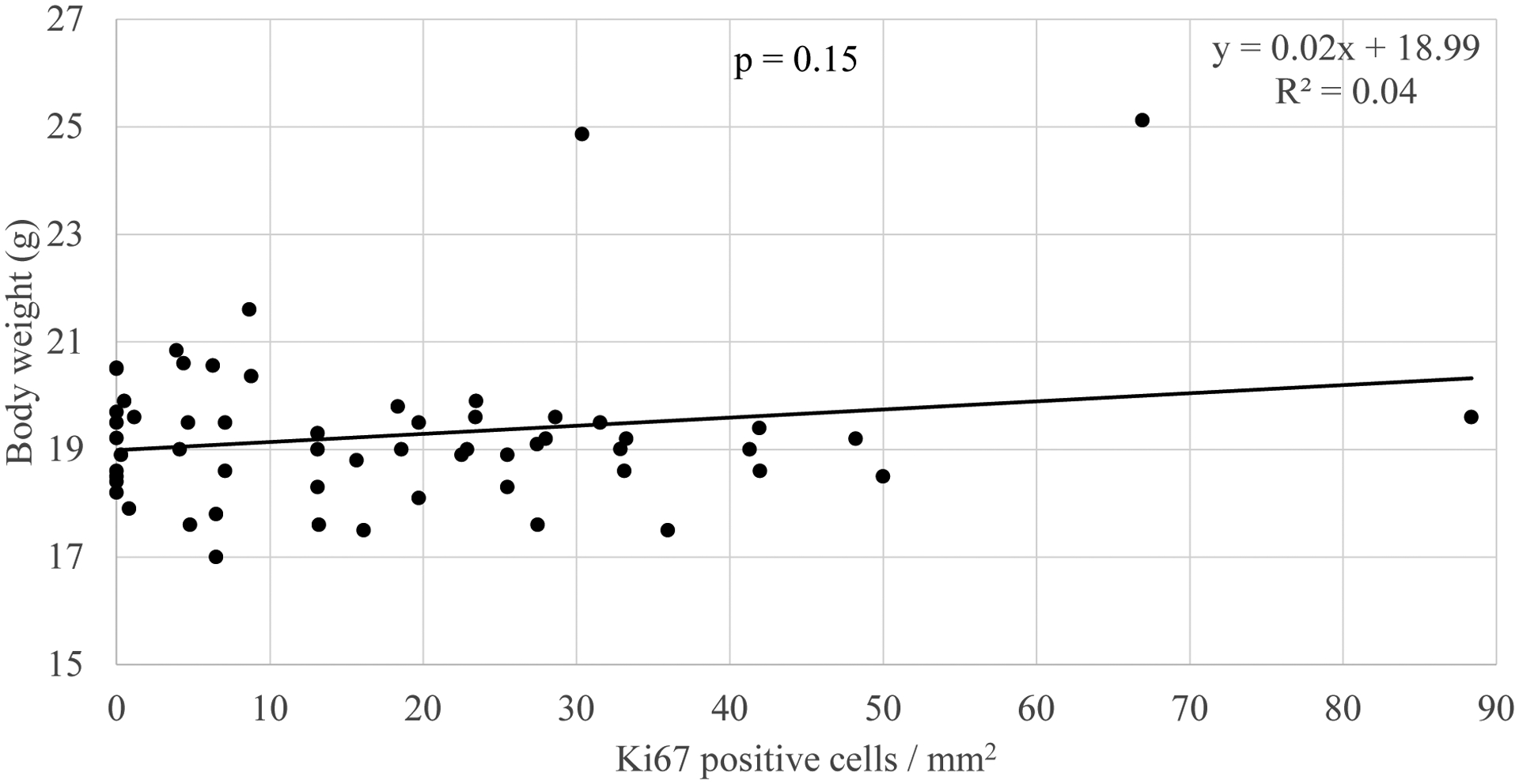
Mammary epithelial cell proliferation compared to body weight in a mouse cohort (n = 58). Body weight of offspring and grandoffspring mice at PND60 following prenatal PAH or negative control exposure compared with Ki67 positive cells in mm^2^ area of the mammary tissue is shown. The linear trendline, its equation, and R^2^ value are shown. Differences in body weight by Ki67 positive cells were analyzed by linear regression.
